# Low amylose content in rice *lowac2* mutant is caused by a new allele of C_2_H_2_ zinc-finger protein that regulates pre-mRNA splicing of *Wx^b^*

**DOI:** 10.1270/jsbbs.25037

**Published:** 2025-11-01

**Authors:** Rina Sakaguchi, Yuko Kozakura, Koume Monma, Naoko Crofts, Hiroyuki Ito, Dong-Jin Kang, Shigeki Hamada

**Affiliations:** 1 Faculty of Agriculture and Life Science, Hirosaki University, 3 Bunkyo-cho, Hirosaki, Aomori 036-8561, Japan; 2 Department of Chemical and Biological Engineering, National Institute of Technology, Akita College, 1-1 Iijima-Bunkyo-cho, Akita 011-8511, Japan; 3 Teaching and Research Center for Bio-coexistence, Faculty of Agriculture and Life Science, Hirosaki University, Gosyogawara, Aomori 037-0202, Japan

**Keywords:** *Oryza sativa*, *dull* mutant, *Waxy* gene splicing, amylose content, starch biosynthesis

## Abstract

The isolated rice mutant, designated *lowac2*, exhibited low amylose content. Whole-genome resequencing and single-nucleotide polymorphism (SNP) fragment analysis revealed that a mutation in the gene encoding the C_2_H_2_ zinc-finger protein in the long-arm terminal of Chromosome 6 was responsible for this low amylose phenotype. An SNP from G to A was observed in the 5ʹ splice junction of intron11, resulting in the production of a protein lacking the C-terminal. Mutation of the C_2_H_2_ zinc-finger protein specifically affected the splicing efficiency of intron1 in *Waxy^b^* (*Wx^b^*). This reduced the levels of granule-bound starch synthase I, which is encoded by *Wx^b^*. Mutations in *lowac2* increased the mRNA expression levels of several starch biosynthetic enzymes, especially starch synthase IIa and starch branching enzyme I. This was consistent with the presence of amylopectin with reduced short glucan chains in *lowac2* seeds compared to wild-type seeds. Furthermore, the crossed lines possessing a gene combination between *Wx^a^* from indica variety and *lowac2* also showed a decrease in amylose content and *Wx^a^* expression; however, this did not affect splicing in *Wx^a^*. The new allele of the C_2_H_2_ zinc-finger protein found in this study affects both *Wx* genes differently.

## Introduction

The improvement of rice quality, along with yield and resistance traits, has become an important breeding goal because it has a direct impact on appearance, taste, milling, and cooking. Starch, which forms water-insoluble granules, is the major storage component in rice endosperm. It is mainly composed of two types of α-glucans: amylose and amylopectin. Amylose constitutes approximately 20%–30% of endosperm starch and is a linear molecule. In contrast, amylopectin accounts for the remaining 70%–80% and is branched (Manners 1989, Preiss and Sivak 1996). In particular, the amylose content of endosperm starch is a primary determinant of the cooking and eating qualities of rice (Tan *et al.* 1999, Webb 1991). Low-amylose rice, whose amylose content is approximately 5%–15%, is often white, turbid, and stickier than normal amylose rice. Starch biosynthesis in rice endosperm involves at least four enzymes: ADP-glucose pyrophosphorylase, soluble starch synthase (SS), starch branching enzyme (SBE), and starch debranching enzyme (Smith *et al.* 1997). In rice, *Waxy* (*Wx*) encodes granule-bound starch synthase I (GBSSI) on Chromosome 6, which is directly involved in amylose synthesis. Quantitative trait locus (QTL) analysis using DNA markers revealed that rice amylose content is regulated in a complex manner by various genes, including *Wx* and *Dull* (*Du*). Two wild-type alleles have been identified: *Wx^a^* of indica and *Wx^b^* of japonica varieties (Sano 1984, Sano *et al.* 1991). The abundance of GBSSI mRNA and protein in the *Wx^a^* allele is approximately 10-fold higher than that in the *Wx^b^* allele, resulting in lower amylose content in japonica than in indica varieties (Zeng *et al.* 2007). Mutations in the *Wx* locus, such as *Wx-mq* and *Wx1-1*, have been identified (Ando *et al.* 2010, Sato *et al.* 2002). In contrast, several *Du* genes, including *Du1* (Satoh and Omura 1981), *Du2*, *Du3* (Satoh and Omura 1986), *Du4*, and *Du5* (Yano *et al.* 1988), modulate the expression of *Wx* to regulate amylose content. For example, *Du1*, which encodes the mRNA splicing factor *Prp1*, reduces *Wx^b^* transcript levels in the endosperm (Zeng *et al.* 2007). Similarly, *Du3* encodes the mRNA cap-binding protein OsCPB20, which forms a cap-binding complex with OsCBP80 and affects the splicing and stability of the *Wx^b^* transcript (Isshiki *et al.* 2000, 2008). However, the genes involved in *Du4*, *Du5*, and other *dull* mutations, such as *du2120* and *du2035* (Kaushik and Khush 1991) are yet to be identified. Several QTLs related to amylose content have been reported (Ando *et al.* 2010, He *et al.* 1999, Li *et al.* 2003, 2011, Septiningsih *et al.* 2003, Takemoto-Kuno *et al.* 2015, Takeuchi *et al.* 2007, Wan *et al.* 2003, 2004). However, the causative genes and mechanisms underlying the regulation of starch biosynthesis in rice remain unclear. Therefore, starch biosynthesis at the genetic level warrants further study.

In this study, we selected low-amylose mutant rice from Tsugaruroman mutant lines, identified a new allele gene encoding a C_2_H_2_ zinc-finger protein that regulates amylose synthesis, and analyzed its effects on *Wx* gene expression.

## Materials and Methods

### Rice plants and mutant lines

All plants were grown in a greenhouse and an experimental paddy field at Hirosaki University, Aomori, Japan. Mature seeds of Tsugaruroman (wild type: WT), a japonica-type cultivar, were treated with 0.1 M phosphate buffer (pH 3.0) containing 1 mM sodium azide to induce mutations (M_1_). Next, the M_1_ seeds were cultivated to harvest self-fertilized seeds (M_2_). The *lowac2* mutant was selected from the M_2_ mutant library based on cloudy appearance and amylose content. The mutants (M_3_) were backcrossed with the WT to generate the segregation progeny F_2_ for next-generation sequencing (NGS) and segregation analysis. Crossing between the F_3_ of the *lowac2* mutant and Hoshiyutaka, an indica-type cultivar, was performed to examine the effect of *lowac2* on *Wx^a^*.

### Observation of seeds and measurement of amylose content

WT and *lowac2* brown rice were horizontally cut in half, and a few drops of potassium iodide solution (2% (w/v) KI and 0.2% (w/v) I_2_) were placed on the cut surface for staining and observation. Scanning electron microscopy (SEM) (JSM-5300; JEOL Ltd., Tokyo, Japan) was used to observe the crystal structure of the endosperm starch granules.

The apparent amylose content was measured using iodine colorimetry as previously described. (Igarashi *et al.* 2021). Polished rice flour was ground in a mortar mill. Next, 100 mg rice flour was mixed with 1 mL of 95% ethanol and 9 mL of 1 M NaOH and left in boiling water for 10 min for complete gelatinization. The volume of the gelatinized solution was adjusted to 50 mL with distilled water. One milliliter of 1 M acetic acid solution and 2 mL of iodide solution (2% (w/v) KI and 0.2% (w/v) I_2_) were added to 5 mL of the gelatinized solution. The volume was subsequently adjusted to 50 mL using distilled water. Absorbance was measured at 620 nm using a spectrophotometer (V-730, JASCO Corp., Tokyo, Japan). The WT (Tsugaruroman with 19% amylose) and two commercially available japonica glutinous varieties without amylose (0% amylose) were used as standards to calculate the apparent amylose content.

### Protein extraction for SDS-PAGE and immunoblotting

Protein samples were prepared as follows. Five hundred microliters of 100 mM phosphate buffer (pH 6.8) was added to five seeds at the developing milky stage (10–15 days after flowering (DAF)). The seeds were mushed for 1–3 min using a Power Masher II (Nippi, Tokyo, Japan). Next, 500 μL of SDS sample buffer (0.1 M Tris-HCl, pH 6.8; 2% SDS; 10% glycerol; 5% 2-mercaptoethanol; and 0.005% bromophenol blue) was added and boiled for 10 min. The mixture was centrifuged at 15,000 *g* for 10 min, and the supernatant was collected and used as a sample (8 μL) for electrophoresis. A 10% gel was used for SDS-PAGE analysis. After electrophoresis, the proteins were transferred onto polyvinylidene fluoride membranes. The membrane was blocked with blocking solution (Blocking One; NACALAI TESQUE, Inc., Tokyo, Japan) and incubated with a diluted anti-GBSSI antibody solution at room temperature for 1 h. It was then incubated with diluted anti-rabbit IgG-labeled alkaline phosphatase at room temperature for 1 h. Color development was performed using 5-bromo-4-chloro-3-indolyl-phosphate and 4-nitro blue tetrazolium solution (Vector Laboratories, Inc., Newark, CA, USA).

### Resequencing and genotype analysis

The genome sequence of *lowac2* was compared with that of the WT using NGS, as previously described (Takahashi *et al.* 2020). Candidate causative genes were subsequently extracted for single-nucleotide polymorphism (SNP) analysis. Mutants were selected based on the amylose content from the F_2_ segregating population of the closing between the WT and *lowac2*. Bulk genomic DNA from the fresh leaves of 10 mutant plants with low amylose content was obtained using a DNeasy Plant Mini kit (QIAGEN, Hilden, Germany). The whole genome of the *lowac2* mutant was resequenced with 48-fold mean coverage using an Illumina HiseqX system (Illumina Inc., San Diego, CA, USA) and aligned based on the rice reference genome (IRGSP1.0) (Kawahara *et al.* 2013). The sequence was compared with previous WT sequence data with 53-fold mean coverage (Takahashi *et al.* 2020). Candidate SNPs were selected based on variant quality (>99.9% degree of confidence) and the SNP index (>0.9). PCR primers for *LowAC2* (C_2_H_2_ zinc-finger protein: Os06g0698859) and probable galacturonosyltransferase 3 (GalUAT: Os06g0727300) genes for SnaPshot fragment analysis were developed and are listed in Supplemental Table 1. The PCR products, including the SNP region amplified with primer pairs OsLowAC2 SNaP-F1/R1 and OsGalUAT SNap-F1/R1, were purified using ISOSPIN PCR Product (NIPPON GENE, Tokyo, Japan). Next, they were subjected to a single-base extension reaction using the SNaPshot Multiplex Ready Reaction Mix (Applied Biosystems, Foster City, CA, USA) according to the manufacturer’s instructions with each specific primer: OsLowAC2 SNaP-F2 or OsGalUAT SNap-F2. The products were subjected to fragment analysis using capillary electrophoresis (3500/3500xL Genetic Analyzer; Applied Biosystems). Primers for cleaved amplified polymorphic sequence (CAPS) markers of the *Wx* allele were used (Igarashi *et al.* 2021). PCR was performed using a DNA template prepared from individual F_2_ plants wherein *lowac2* and Hosiyutaka were crossed, 1 pmol of each primer pair, and KOD FX Neo (TOYOBO, Osaka, Japan). PCR products were digested using *Acc*I (TaKaRa Bio Inc., Shiga, Japan). The reaction solution was separated on 1% agarose gel and visualized using Gel Green (FUJIFILM Wako Pure Chemical Corp., Tokyo, Japan) to determine the genotype.

### RT-PCR and real-time PCR analysis

Total RNA of *lowac2* mutants and the WT was extracted from seed endosperms harvested approximately 15–20 DAF using RNAzol RT (Molecular Research Center Inc., Cincinnati, OH, USA). First-strand cDNA was synthesized using the PrimeScript RT MIX (TaKaRa) for reverse transcription. The synthesized reverse transcription products were used as templates for real-time PCR. The PCR primers for RT-PCR and real-time PCR of *lowac2* and *waxy* rice are listed in Supplemental Table 1. RT-PCR for OsGBSSI was performed using the primer pairs OsGBSSI-F1/R1, -F2/R2, and -F3/R3. OsLowAC2 SNaP-F1 was also used for RT-PCR of the 3ʹ region on *LowAC2* with OsLowAC2 RT-R1 primer. Other gene expression profiles of enzymes related to starch biosynthesis were determined using the same method and primer pairs as previously reported (Igarashi *et al.* 2021). The mRNA expression level of ubiquitin (Os03g0234200) was used as an endogenous control, and the relative expression level was calculated using the ΔΔCT method and compared with the WT set as 1.0.

### Amylopectin chain length distribution

Chain length distribution was analyzed using fluorophore-assisted capillary electrophoresis (FACE) as previously described (Fujita *et al.* 2001). *Pseudomonas amyloderamosa* isoamylase (Nagase Viita Co., Ltd., Okayama, Japan) was used to debranch amylopectin. Fluorescent labeling of debranched glucan chains was performed using a PA800-plus carbohydrate labeling and analysis assay kit (Beckman Coulter, Brea, CA, USA), followed by capillary electrophoresis using a P/ACE MDQ Plus capillary electrophoresis system (AB SCIEX, Tokyo, Japan), according to the manufacturers’ instructions.

## Results

### Evaluation of grain phenotypes

**Fig. 1.**
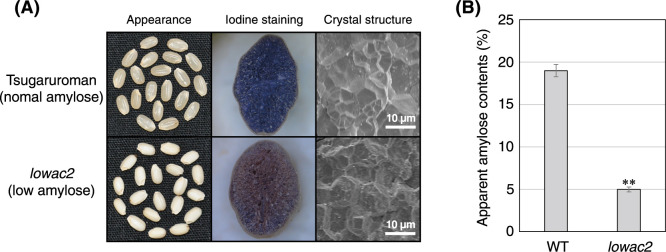
Comparison of phenotypes and amylose content between the WT and *lowac2* mutant. (A) Left panel, appearance of mature dehulled grains; middle panel, I_2_-stained cross-sections of maturing grain; right panel, electron microscope visualization of the transverse section of rice grains under 500× magnification. (B) Amylose contents of mature seeds of the WT and *lowac2*. Data are expressed as the mean ± standard error (SE; n = 3) (**P < 0.01).

The rice *lowac2* mutant was selected based on its cloudy appearance and low-amylose phenotype. The heading date and plant height of *lowac2* were almost the same as those of Tsugaruroman. The brown rice of *lowac2* grains were slightly smaller, and the 1,000-grain weight was 22.0 g and 19.1 g for Tsugaruroman and *lowac2*, respectively. A comparison of the grain morphology and amylose content between Tsugaruroman (WT) and *lowac2* is shown in Fig. 1A. The *lowac2* mutant showed white turbidity in appearance. WT and *lowac2* were stained blue-purple and red-purple, respectively, when cross-sections of the seeds were stained with iodine. This suggests that the selected *lowac2* had a low amylose content. The amylose contents of the WT and *lowac2* were 19.0% and 5.0%, respectively (Fig. 1B). The crystal structures of the starch granules observed by SEM were similar in both the WT and *lowac2*, with a densely packed structure.

### Expression of GBSSI protein and *Waxy^b^* gene in *lowac2* seeds

**Fig. 2.**
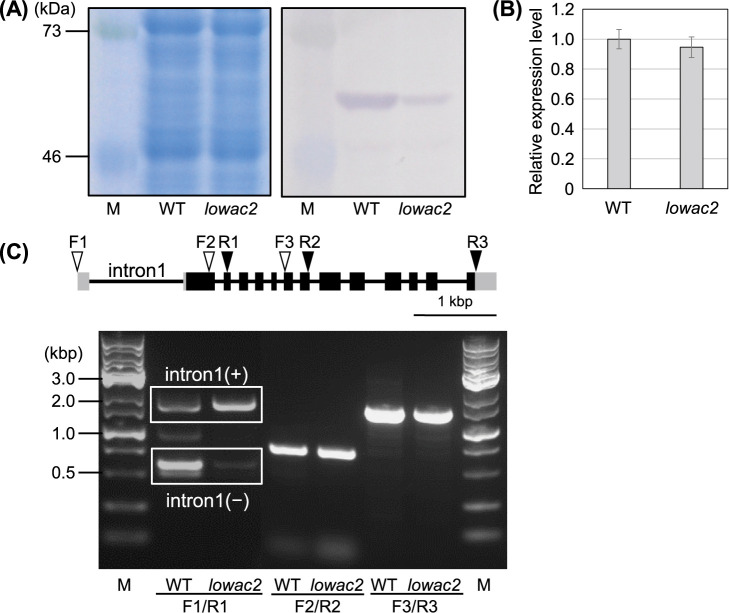
Expression and splicing of *Wx^b^* in the *lowac2* mutant. (A) SDS-PAGE (Left panel) and immunoblotting (Right panel) results obtained using antiserum raised against purified recombinant GBSSI protein. (B) Real-time PCR analysis of *GBSSI* transcript levels in the WT and *lowac2* mutant. The transcript level of the WT was set to 1. Data are expressed as the mean ± SE (n = 3–5). (C) Schematic structure and RT-PCR analysis of *Wx^b^*. The UTRs, exons, and introns are depicted as gray boxes, black boxes, and solid lines, respectively. The opened and closed arrowheads in the genetic structure indicate the position of sense and antisense primers for RT-PCR, respectively. RT-PCR of the splicing efficiencies of *Wx^b^* using three primer pairs: OsGBSSI-F1 (F1) &-R1, OsGBSSI-F2 (F2) & -R2, and OsGBSSI-F3 (F3) & -R3.

Western blotting using a GBSSI-specific antibody revealed that the level of GBSSI expression in *lowac2* was significantly lower than that in the WT (Fig. 2A). However, the expression of GBSSI mRNA was the same in both the WT and *lowac2*, suggesting that the reduced GBSSI protein expression in *lowac2* was due to post-transcriptional control (Fig. 2B). Therefore, the structure of the *Waxy^b^* (*Wx^b^*) transcript encoding GBSSI was confirmed. *Wx^b^* often exhibits reduced GBSSI protein levels and low amylose contents owing to alternative splicing of the first intron (Cai *et al.* 1998, Isshiki *et al.* 1998, 2000). Transcripts that retain the first intron cannot translate the GBSSI protein, and only normal transcripts without introns are translated. RT-PCR results showed that normal transcripts without the first intron, as showed two types of products called cite1 and cite2 (Igarashi *et al.* 2021), were significantly reduced in *lowac2* compared to the WT, and that transcripts containing the first intron were increased. In contrast, the other regions were spliced normally and showed no differences from the WT (Fig. 2C).

### Identification of causative genes

**Fig. 3.**
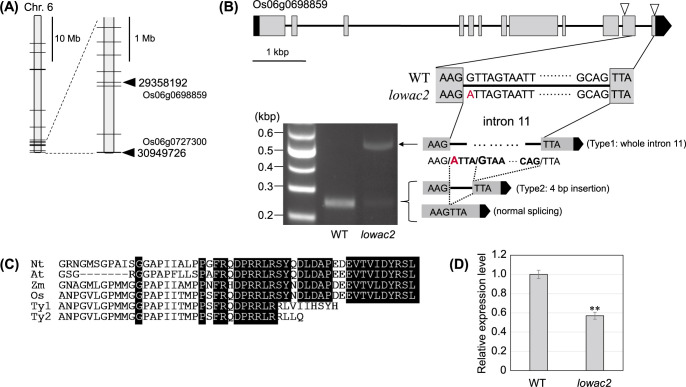
Analysis of the causative gene for the *lowac2* mutant. (A) SNP information of the *lowac2* locus, as determined through whole-genome sequencing. The horizontal lines on the chromosome bars indicate SNP positions. The two arrowheads indicate candidate genes harboring an SNP in an ORF. (B) Gene structure of C_2_H_2_ zinc-finger protein and mRNA products of SNP. The UTRs, exons, and introns are depicted as gray boxes, black boxes, and solid lines, respectively. Two arrowheads in the genetic structure indicate the position of forward and reverse primers for RT-PCR. The RT-PCR products were analyzed on agarose gel. The position of the red letter shows SNPs with G to A mutation in *lowac2*. The dashed lines indicate splicing patterns. (C) Aliments of the C-terminal region of C_2_H_2_ zinc-finger protein-related sequences from several plants. Nt, *Nicotiana tabacum*; At, *Arabidopsis thaliana*; Zm, *Zea mays*; Os, *Oryza sativa*; Ty1 and Ty2, alternative splicing from Type1 and Type2 in this study. (D) Transcript levels, as determined through real-time PCR of C_2_H_2_ zinc-finger proteins in the WT and the *lowac2* mutant. The transcript level of the WT was set to 1. Data are expressed as the mean ± SE (n = 3–5) (**P < 0.01).

Comparison of whole-genome resequencing analyses of Tsugaruroman and *lowac2* identified a total of 64 mutations (32 SNPs and 32 indels). Most mutations were located in non-coding regions, and only two SNPs were identified in the ORF. Both SNPs were G-to-A substitutions located at the end of the long arm of Chromosome 6. The two genes were identified as the C_2_H_2_ zinc-finger protein gene (Os06g0698859) and probable galacturonosyltransferase 3 (OsGalUAT3: Os06g0727300) (Fig. 3A). The SNP index was 1 (37/37) for the C_2_H_2_ zinc-finger protein and 0.93 (28/30) for OsGalUAT3. To identify the causative gene, genotype-phenotype correlations were examined using fragment analysis of the SNapShot method for 43 individuals with normal amylose properties and 11 individuals with low amylose properties from the F_2_ segregating population (Supplemental Table 2). For the C_2_H_2_ zinc-finger protein, all SNP genotypes in individuals with normal amylose intake were either WT homozygous (GG) or heterozygous (GA). All SNPs were mutant homozygous (AA) in low-amylose plants. For OsGalUAT3, one homozygous (AA) and two heterozygous (GA) mutants were observed in normal and low-amylose individuals, respectively. This confirmed the mismatch between the genotype and phenotype. Furthermore, in the backcrossed F_2_ segregating generation, the normal and low amylose trait segregated at a ratio of approximately 3:1 (observed values, 45:16; calculated *p*-value for the chi-square test, 0.82), suggesting that the cause of low amylose is a recessive gene encoding the C_2_H_2_ zinc-finger protein. Based on these findings, the causative gene of *lowac2* was identified as the C_2_H_2_ zinc-finger protein gene.

The SNP that replaced G with A in the C_2_H_2_ zinc-finger protein gene of *lowac2* was observed in the 5ʹ splicing recognition site of Intron 11 (Fig. 3B), suggesting that this intron may remain in the transcript. We performed RT-PCR using primers that sandwiched the 11th intron region and obtained a longer PCR product for the C_2_H_2_ zinc-finger protein gene of *lowac2* (Type 1). Sequence analysis of this amplified fragment confirmed the insertion of Intron 11. Furthermore, a faint PCR product in *lowac2* was observed at the same position as that in the WT on the gel. The sequencing results revealed two different splicing products in the PCR products. One included an additional four bases downstream of the original splicing position, which was generated by recognizing the GT four bases downstream as the splicing site (Type 2). The other form was the same normal-spliced product as that of the WT. These incomplete products (Types 1 and 2) immediately generated stop codons owing to frameshifts (Fig. 3C). In addition, the mRNA level of C_2_H_2_ zinc-finger protein in *lowac2* was 0.56-fold lower than that in the WT (Fig. 3D).

### Expression levels of starch synthesis-related genes

**Fig. 4.**
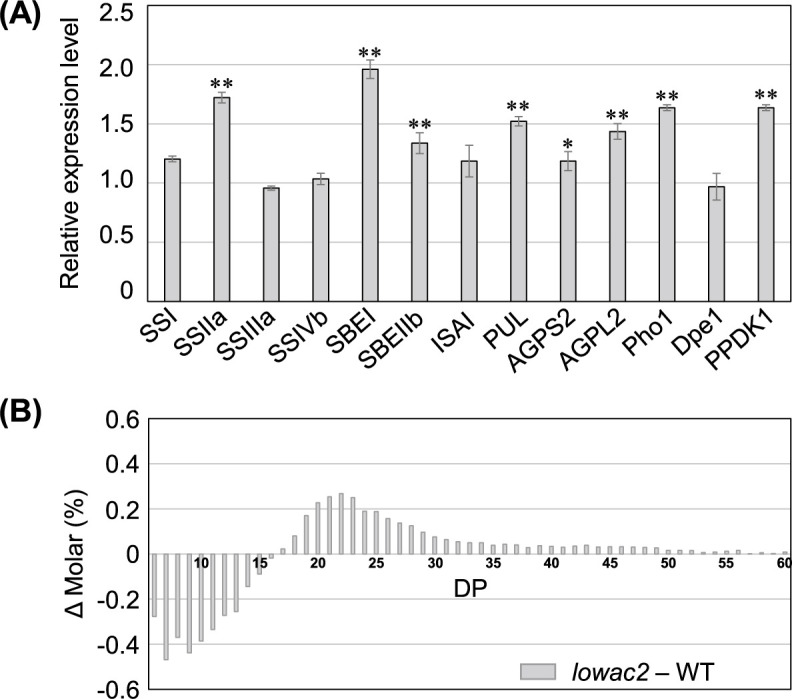
Effects of the *lowac2* mutation on the expression of genes involved in starch biosynthesis and on amylopectin glucan chain distribution. (A) The expression levels of starch biosynthesis-related genes in the WT and *lowac2* mutants, as determined using real-time PCR. The expression levels of all target genes relative to the WT transcript level are shown. Error bars indicate standard error (n = 5) (*P < 0.05, **P < 0.01). (B) Chain length distribution patterns of endosperm amylopectin in WT and *lowac2* mature seeds, as determined using the FACE method. The result indicates differences in chain length distributions between the *lowac2* mutant and the WT.

In addition to GBSSI, various enzymes are involved in starch biosynthesis. Real-time PCR analysis showed that the mRNA expression levels of starch biosynthetic enzymes in *lowac2* was higher than those in the WT (Fig. 4A). In particular, the expression levels of SSIIa and SBEI mRNA in *lowac2* were 1.73- and 1.96-times higher than those in the WT, respectively. These changes in gene expression may affect the amylopectin structure. Analysis of the chain length distribution of glucan units in amylopectin of the WT and *lowac2* showed that amylopectin from *lowac2* contains relatively fewer short chains (degree of polymerization <17) and more middle chains (degree of polymerization ≥17) than that from the WT (Fig. 4B).

### Effects of *lowac2* on the *Waxy* allele

**Fig. 5.**
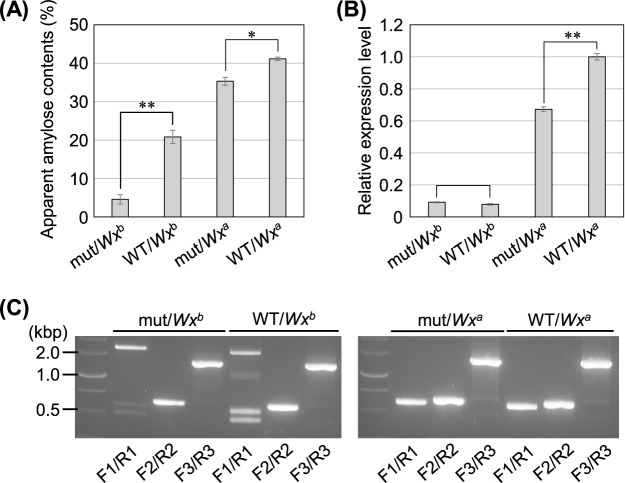
*Waxy* allele specificity of the C_2_H_2_ zinc-finger protein. (A) Comparison of apparent amylose contents in four classes of lines with different genotypes. Error bars indicate standard error (n = 4–6) (*P < 0.05, **P < 0.01). (B) The expression levels of *Wx* genes in different genotype lines. The expression levels relative to the *LowAC2*/*Wx^a^* transcript level are shown. Error bars indicate standard error (n = 3) (**P < 0.01). (C) RT-PCR analysis for the splicing efficiencies of *Wx* genes. The same three primer sets (F1/R1, F2/R2, and F3/R3) as in Fig. 2C were used to amplify *Wx* regions.

To elucidate the specificity of the C_2_H_2_ zinc-finger protein on the *Waxy* allele (*Wx^a^* and *Wx^b^*), the *lowac2* mutant was crossed with the indica cultivar Hoshiyutaka, which carries *Wx^a^* and has a high amylose content. The genotypes of *LowAC2* and *Waxy* in 125 individuals of the F_2_ generation were analyzed using the SNaPShot method and CAPS markers, respectively. Individuals with different genotype sets for both genes were selected, including four *lowac2*/*Wx^b^* lines, four *LowAC2*/*Wx^b^*, six *lowac2*/*Wx^a^* lines, and four *LowAC*2/*Wx^a^* lines. Amylose content was measured using F_3_ seeds of self-pollinated lines from the selected F_2_ individuals; the average amylose content was 4.6%, 20.8%, 35.3%, and 41.2% in *lowac2*/*Wx^b^*, *LowAC2*/*Wx^b^*, *lowac2*/*Wx^a^*, and *LowAC*2/*Wx^a^* lines, respectively (Fig. 5A). The expression levels of *waxy* genes were analyzed for each genotype combination. As previously reported, *Wx^b^* lines showed approximately 1/10 lower expression levels than *Wx^a^* lines (Zeng *et al.* 2007). Furthermore, the *lowac2* mutant showed approximately 70% lower expression even among the *Wx^a^* lines (Fig. 5B).

We also evaluated the splicing of each *Waxy* mRNA using RT-PCR (Fig. 5C). In *Wx^a^*-harboring individuals, neither abnormal splicing of the first intron observed in *Wx^b^*-harboring individuals nor the splicing abnormalities were observed in other regions. These results indicate that the identified C_2_H_2_ zinc-finger protein specifically acts on the splicing of *Wx^b^* alleles.

## Discussion

The *dull* mutant, which regulates GBSSI expression, reduces the amylose content of rice endosperm starch. Various *dull* genes involved in the regulation of pre-mRNA splicing of *Wx^b^* have been identified in previous studies, demonstrating the complexity of the regulatory mechanism underlying GBSSI expression. However, the mechanisms involved in starch biosynthesis are yet to be elucidated; therefore, the starch biosynthetic pathway warrants further study. In this study, we selected *lowac2*, which exhibits low amylose traits, from the Tsugaruroman mutant line, identified its causative gene, and performed functional analysis.

We identified the causative gene for *lowac2* as a C_2_H_2_ zinc-finger protein located on Chromosome 6. In previous reports, a C_2_H_2_ zinc-finger protein null mutant that is the same gene as *LowAC2* exhibited low amylose contents (Cai *et al.* 2022, Zhao *et al.* 2022). This result strongly suggests that the causative gene for *lowac2* is also a C_2_H_2_ zinc-finger protein and indicates that *lowac2* is a novel allele of this gene.

The expression of C_2_H_2_ zinc-finger protein mRNA in *lowac2* was reduced to approximately half that in the WT (Fig. 3D). Because the primers for real-time PCR were designed at the upstream of the SNP site, the expression results include both splicing-normal and abnormal mRNA. However, RT-PCR showed extremely low amounts of lowac2 normal mRNA (Fig. 3B), suggesting that most of the mRNA is abnormal splicing mRNA. Although the cause is unclear, intron insertion due to SNP may destabilize the structure of the C_2_H_2_ zinc-finger protein mRNA. Thus, the C_2_H_2_ zinc-finger protein in *lowac2* lacks 20 amino acids in its C-terminus because of intron insertion. Despite the short deletion of the C-terminus, *lowac2* exhibited low amylose contents comparable to those of a previously reported null mutant with a stop codon in exon 8 (Cai *et al.* 2022, Zhao *et al.* 2022), suggesting that the C-terminus of the C_2_H_2_ zinc-finger protein plays an important role in its function. However, whether the loss of function of the C_2_H_2_ zinc-finger protein in the *lowac2* mutant is due to the loss of function of the C-terminus via intron insertion or due to a quantitative effect caused by the reduction in the amount of mRNA remains unclear.

C_2_H_2_ zinc-finger proteins mediate the cap-binding complex and spliceosome during pre-mRNA processing and indirectly affect splicing (Montgomery and Carrington 2008). This suggests that the C_2_H_2_ zinc-finger protein encoded by the mutation-causing gene in this study may also be involved in GBSSI mRNA splicing, which is responsible for amylose synthesis, resulting in low amylose content in *lowac2*. Our data revealed that the causative gene of *lowac2* affects the alternative splicing of the first intron of *Wx^b^* pre-mRNA. These results are similar to those for *du1*, *du3*, and *lowac1*, which have been identified as causative genes for low amylose content. This suggests that the C_2_H_2_ zinc-finger protein identified in this study also contributes to the alternative splicing of *Wx^b^* by interacting with other proteins. However, future studies are required to analyze protein-protein interactions using yeast two-hybrid assays and other methods.

SSIIa and SBEI mRNAs were significantly more highly expressed in the *lowac2* mutant than in Tsugaruroman. SSIIa elongates the amylopectin chains from DP8 produced by SSI to produce chains up to approximately DP24, creating the middle glucan chains of amylopectin (Nakamura *et al.* 2005). SBEI is an effective enzyme for the transfer of long glucan chains and contributes to the synthesis of middle- and long-chain branches (Nakamura 2002). The increased expression of these enzymes suggests that the middle and long chains of amylopectin may be increased in *lowac2*. Analysis of the chain length distribution of amylopectin revealed a decrease in the proportion of short chains and an increase in the proportion of medium and long chains in *lowac2* compared to Tsugaruroman, which is consistent with the results predicted from the gene expression analysis. This suggests that the C_2_H_2_ zinc-finger protein-encoding gene may affect not only amylose content but also the molecular structure of amylopectin. Because C_2_H_2_ zinc-finger protein is a splicing factor, it may be involved in the regulation of gene expression in not only GBSSI but also many other genes. In fact, there is a report that it is involved in various miRNA processing (Cai *et al.* 2022). In addition, another GBSSI splicing factor that shows low amylose property also shows an increase in the expression of starch synthesis genes (Igarashi *et al.* 2021). These suggest that C_2_H_2_ zinc-finger protein is directly involved in the expression of other starch synthesis genes, but do not rule out the indirect effect of altered expression of GBSSI.

We investigated whether the C_2_H_2_ zinc-finger protein acts similarly to the *Wx* alleles *Wx^a^* and *Wx^b^*. The mut/*Wx^b^*, a genotype similar to that of *lowac2*, showed low amylose content, which were also reproducible. Furthermore, a significant difference in amylose content was also observed between mut/*Wx^a^* and WT/*Wx^a^*. However, no splicing abnormalities were observed in mut/*Wx^a^*, suggesting that splicing regulation by the C_2_H_2_ zinc-finger protein is specific to *Wx^b^* and the splicing abnormalities depends on the SNP sequence between GBSSI alleles. Although the reason for this is unclear, the decrease in C_2_H_2_ zinc-finger protein function may affect the stability of *Wx^a^* mRNA and/or transcription level of *Wx^a^*.

In this study, we successfully identified a novel allele of the C_2_H_2_ zinc-finger protein involved in GBSSI mRNA splicing and amylopectin synthesis.

## Author Contribution Statement

SH designed the research. RS, YK, KM, NC, IH and SH performed the investigations. DJK contributed glowing mutant lines. SH wrote the manuscript. IH reviewed and edited the manuscript. All authors have read and approved the final version of the manuscript.

## Supplementary Material

Supplemental Tables
