# Supplementary material for: Low amylose content in rice lowac2 mutant is caused by a new allele of C2H2 zinc-finger protein that regulates pre-mRNA splicing of Wxb
Source: Breed Sci. 2025 Nov 1;75(5):421–9. doi: 10.1270/jsbbs.25037 (PMC13129570; doi:10.1270/jsbbs.25037)
Supplement: Supplementary file 1 — Supplemental Tables [file 75_421_s1.pdf]

doi: 10.1270/jsbbs.25037

Supplemental Table 1. List of primers used in this study

Primer name	Sequence (5′-3′)
For SNaPshot	
OsLowAC2 SNaP-F1	ATTTGTTCCTGCACCTCCAGAAATAGC
OsLowAC2 SNaP-F2	AGATCCTCGCCGTTTGCGAAG
OsLowAC2 SNaP-R1	ATCCAAGCAAAATAGGAGAATAGA
OsGalUAT SNaP-F1	AAATATCGATAGTGTTAAGAAGCAGAA
OsGalUAT SNaP-F2	GGAGGAAGAAGATAAGGCAAAGCGTGAG
OsGalUAT SNaP-R1	TAACAACTACTGAGGCTGCAAGGACGT
For RT-PCR	
OsGBSSI-F1	ACCATTCCTTCAGTTCTTTGTCTATCTCA
OsGBSSI-F2	AACGTCGTGTTCGTCGGCGCCGAGATG
OsGBSSI-F3	GTGAGGATGTTGTGTTCGTCTGC
OsGBSSI-R1	TGTACTGGTCGTACCGAGGAGAG
OsGBSSI-R2	CGTCGATGAAATCGAAGGATGACCTGAAC
OsGBSSI-R3	CAGGGTCCGATAAGATACTTAAGCACACCC
OsLowAC2 SNaP-F1	ATTTGTTCCTGCACCTCCAGAAATAGC
OsLowAC2 RT-R1	CAAACTTCTGTAGTCAAGAACG
For real-time PCR	
OsLowAC2 qPCR-F1	CTGAGATATGACCGTGCCAAT
OsLowAC2 qPCR-R1	CACCAGGAATAGGCATCAAAA
OsPho1 qPCR-F1	ACGATGACTTGATGGGTTCTCT
OsPho1 qPCR-R1	GCGGTATGCTTTATCAACCTTCT
OsDpe1 qPCR-F1	GCCTGAGGCTAATAGGACTGAA
OsDpe1 qPCR-R1	TCCCTTTCTGTGTAGCTGGAGT
OsPPDK1 qPCR-F1	AACCATGACCAACCAGGGTA
OsPPDK1 qPCR-R1	TGGCAATTTGACGGATAACA

Supplemental Table 2.

Correlation between amylose phenotype and genotype of candidate genes. Individuals in red letter show no correlation with the phenotype. Lines with normal amylose are shown as part of the results of a total of 43 individual analyses. A: Mutant homozygous, G: WT homozygous, GA: Heterozygous.

nomal amylose	1	2	3	4	5	6	7	8	9	10	11	12	13	14	15	16	17	18	19	20	21
Os06g0698859	G	G	GA	G	G	GA	G	GA	GA	G	G	G	GA	GA	GA	GA	G	G	GA	G	GA
Os06g0727300	G	GA	GA	GA	G	GA	G	GA	GA	G	G	G	GA	GA	GA	G	G	G	GA	G	A

low amylose	1	2	3	4	5	6	7	8	9	10	11
Os06g0698859	A	A	A	A	A	A	A	A	A	A	A
Os06g0727300	A	GA	GA	A	A	A	A	A	A	A	A
